# Can earthen architectural heritage save us?

**DOI:** 10.1186/s43238-021-00041-x

**Published:** 2021-11-10

**Authors:** Sebastien Moriset, Bakonirina Rakotomamonjy, David Gandreau

**Affiliations:** Centre international de la construction en terre, Ecole Nationale Supérieure d’architecture de Grenoble, CRAterre, CS 12636 - 38036 GRENOBLE, Cedex 2, France

**Keywords:** Learning from the past, Endogenous knowledge, Earthen architecture, Sustainable architecture

## Abstract

Contemporary architecture seems to turn its back on the past in terms of the raw materials taken from the environment, their transformation into building components and the way they are assembled to create buildings. The global challenge of preserving the environment forces us to rethink the way we produce architecture today. Within this challenge, the past shows us possible ways to fill the gap between tradition and modernity. However, we need to understand what motivates people to abandon ancestral materials and knowledge for materials that they cannot manufacture or use themselves. Is this transformation to industrial materials and forms irreversible? Is there nothing we can learn from our rich past? How can we revive endogenous knowledge to produce environmentally wise architecture? These are the questions that the authors, who have been working on the revival of earthen heritage for over 20 years, wish to answer.

## Introduction

Living in the 21st century confronts us with the challenge of rethinking our lifestyles so as not to destroy the planet that hosts us. Planet earth is like vernacular earthen architecture: seriously disfigured by a lack of consideration and protection. Contemporary architecture turns its back on endogenous knowledge and is a major contributor to environmental disruption. It relies on standardised building systems using industrial materials derived from natural resources extracted at locations far away from the building sites. It is uprooted from the environment in which it is built; it disregards the social links between inhabitants and craftsmen as well as the cultural image it conveys and the economic resilience of the territories it occupies. Genuine vernacular architecture, the result of centuries of successive improvements based on what the surrounding landscape has to offer, still exists in some places, but it is threatened with extinction everywhere, as the transmission of knowledge necessary for its survival has become difficult. Seeing traditional craftsmen at work is possible only where people have no other choice, as their income does not give them access to so-called modern materials. Beautiful stone or earthen architectures that seem to emerge naturally from the ground are becoming rare. But why are they disappearing? What motivates people to abandon ancestral knowledge for building materials that they cannot manufacture or use themselves? Is this transformation to industrial materials and forms irreversible? Is there nothing we can learn from our rich past? These are the questions that the authors, who have been working on the revival of earthen heritage for over 20 years, wish to answer.

## Reasons for the abandonment of vernacular earthen architecture

The greatest admirers of vernacular earthen architecture are not necessarily its inhabitants but researchers or external visitors who admire the designs’ originality and the constructive intelligence and praise the thermal comfort of these constructions. They are indeed right to be impressed by such advanced knowledge, the result of centuries of cumulative improvements and collective creativity. However, the inhabitants of these structures do not see this architecture in the same light and aspire to live somewhere else.

They consider with little excitement these familiar models that hardly meet the needs that have evolved. These aspirations for change are motivated by three main ideas: the rejection of archaic models, the desire to eliminate maintenance and the wish for a “modern” life in the image of globalised models.

### Rejecting archaism

**Fig. 1:**
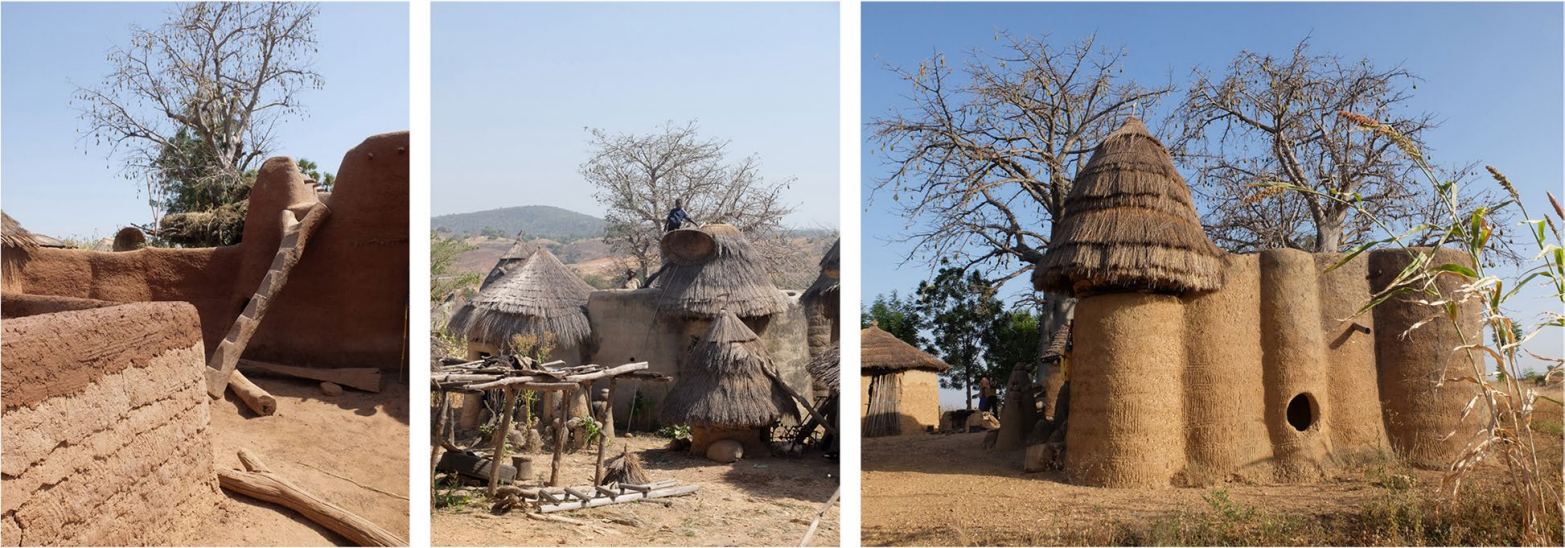
Architecture of the Koutammakou cultural landscape, Benin (Source: the authors/CRAterre)

Vernacular architecture suffers from archaism because it conveys the image of an outdated way of life. Living in a traditional earthen house means continuing to live as many previous generations did, according to the rhythm of the seasons and in osmosis with the material, cultural and climatic realities of the place. It is true that these architectures are often rigidly tied to models that are difficult to modify. The very beautiful fortified earthen houses of Koutammakou, for example (North Benin and North Togo), thousands of examples of which still inhabit the landscape, are built according to only 5 models, which correspond to a meticulously regulated secular organisation of life. In these beautiful and complex monolithic constructions (Fig. 1), each space has its own function, linked to specific activities of the day and night and each of the seasons. When a person is born in one of these houses, he or she is part of a conception of life ordered by his or her ancestors, which functions and ensures his or her survival but which is unfortunately at odds with the canons of modern idle life conveyed by the media. The relevance of these architectures is obvious to elderly individuals, but it raises questions for younger generations. Traditional homes cannot adapt to changes in lifestyle by, for example, integrating new furniture or household appliances. However, they are the ideal houses for an ancestral way of life forged through a strong link between humans and the environment that reinforces resilience but remains impervious to external influences. These architectures are admired by researchers because they positively meet most of the sustainable development criteria,^1^ but their inhabitants do not see it that way and are looking for a radical change in direction.

### Eliminating maintenance

Another weakness of vernacular architecture is the maintenance burden it imposes. It is undeniable that earthen architecture survives through cyclical maintenance protocols that have been applied for centuries. But do not other modern materials also require maintenance? The centuries-old earthen architectures that can be admired today owe their survival to traditional maintenance practices that are an integral part of these architectures: they are built with fragile available resources, but their weaknesses are overcome with appropriate architectural details and cyclical, often annual, care. This approach to controlled fragility is abhorred by the “hard” architecture that is promoted by regulations and standards, i.e., governments. Building with earth has even become illegal in some places. The drudgery of repeated maintenance often leads owners to abandon their properties and switch to “hard” construction. In other cases, they look for alternatives to protect these earthen surfaces by applying sand and cement plasters, for example. While this works in some cases, these hybrids often fail and encourage only the rejection of earth. This shift towards other building techniques is sometimes also dictated by the difficulty of mobilising human resources or finding materials such as straw for roofing, which is no longer as abundant as it was due to changes in land use.

### Living a modern life

One of the weaknesses of humans is our permanent dissatisfaction. We perceive the lifestyles and architectures of other countries through films, TV series and the media in general. This visibility creates envy and a desire for a less laborious life in “modern” architecture, with large, bright, sanitised rooms. Traditional mud houses are often the very anthesis of this vision, offering dark spaces of small dimensions limited by the length of the wooden beams in the floors and roofs. These vernacular architectures are often linked to an entirely outdoor life during the day, punctuated by a series of intense physical labour that contrasts with the more comfortable indoor lifestyle sold by the media.

### Loss of knowledge

Another reason for the decline in earthen architecture is the loss of know-how: it is of course laudable to offer training to younger generations and to open the doors of technical schools offering diplomas in carpentry, masonry or plastering, but these schools promote a globalised vision of architecture imposed by industrial standards. Regardless of the continent, training is a problem because it first makes a selection of options and systematically rejects artisanal knowledge and know-how as well as traditional materials, techniques and shapes. That freedom of the hand that allows the worker to perfect him or herself and forge his or her autonomy has disappeared (Ferro 2005). At the dawn of the 21st century, it is still difficult to find training courses in architecture that are open to all options, including those developed locally over centuries. However, it is by mastering all the options that an architect or builder will reach the best compromises. In the absence of a wide range of knowledge, the young graduate will apply what he or she has learned, which is mostly to assemble prefabricated building elements from industry according to standardised implementation protocols. This approach ultimately makes creativity and hybridisation difficult.

## Restore the desire to build with earth

This gradual abandonment of earthen materials should not be seen as inevitable. Other parameters allow us to modify this pessimistic vision of the future of earthen architecture. The growing mobilisation of governments to find political solutions to help save the planet (from the 1987 Brundtland Report to the Earth Summits that followed) and to consider our lifestyles in a more ecological way is indeed stimulating a young generation of designers who are trying to recapture the benefits of local resources. Various examples of environmentally virtuous architectures are emerging around the world, some using earth as the main material. Building a resolutely contemporary architecture integrating endogenous knowledge has become a reality. However, the challenge of revitalising earthen architecture remains immense. Several approaches are available to meet this challenge.

### Who is building with earth today?

**Fig. 2:**
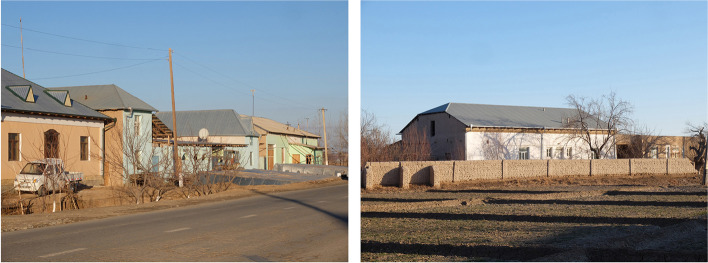
Contemporary earthen houses in Uzbekistan (Source: the authors/CRAterre)

Of the millions of people who still build with earth today, we can easily distinguish 3 main profiles of builders. The first profile, the most numerous, includes those who do not have the means to build in any material other than earth. They sometimes build in a sloppy manner and often do not maintain their property. Considering their construction to be a temporary option, they dream of 1 day having access to a proper “hard” building. These careless constructions damage the image of earthen constructions and encourage the rejection of the material. The second profile includes those who keep their vernacular heritage alive. As their ancestors did, they utilise what they were taught by tradition, making small changes each time. However, they keep using the earth. This is the case, for example, in Uzbekistan (Fig. 2), where brand new, well-built houses made of cob or adobe are omnipresent in small towns. The architecture has evolved to incorporate larger openings than in the past, yielding beautiful, bright and pleasant interior spaces. However, the endogenous spirit of doing the best with the smallest amount of resources remains unchanged.

**Fig. 3:**
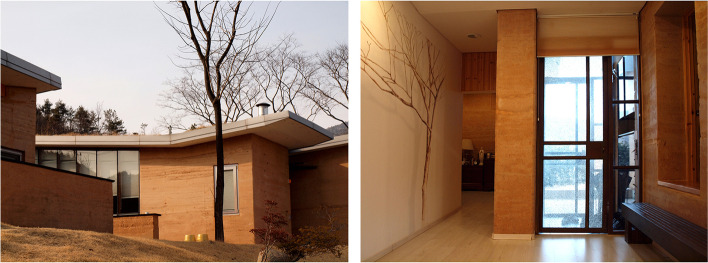
Contemporary earthen houses in Korea, architect Yilwoo Lee (Source: the authors/CRAterre)

The last profile, the least numerous, includes people who have the financial capacity to access all the material options offered by the market and who choose earth on purpose, for a variety of reasons: sanitary, aesthetic, ecological, etc. This category is developing very quickly and suggests that earth is no longer just a material for the poor but is also a valuable material in demand for luxury buildings (Fig. 3). These people are ready to pay extra to avoid the conventional houses imagined by industrial material dealers.

### Finding hybrid models and returning to endogenous knowledge

In 1964, the exhibition “Architecture without an Architect” at the MoMA in New York, created by architect Bernard Rudofsky, highlighted the constructive genius of many of the world’s populations (Rudofsky 1964). This event had a global reach because the architectures presented reflected an age-old tradition that has constantly regenerated itself in response to social and environmental changes.

Several activist architects have supported the revival of vernacular architecture. While the work of Hassan Fathy serves as an historical reference (Fathy 1973), internationally renowned contemporary architects are also involved in such work. Such architects demonstrate that multiple strategies for the revival of vernacular earthen architecture are possible.

Since the beginning of the 21st century, a Burkinabe architect, Francis Kéré, has been constructing community buildings out of earth: primary and secondary schools, public libraries, women’s centres, and medical and cultural centres. His work provides essential responses to communities’ needs. Through these buildings, he helps restore a positive image of this material, which is considered poor and obsolete in these communities. His architecture is generous in its function and of high quality. Kéré reinterprets vernacular wisdom by grafting his innovative creativity onto it. Finally, like his visionary compatriot Thomas Sankara, he supports short supply chains that reinforce the local economy (Guillaud 2019).

The work of Anna Heringer, an Austrian architect who favours earth and other materials collected on building sites, inspires the reappropriation of vernacular architecture for its common sense and for the social and cultural continuity it offers. Through her projects, such as the famous METI school in Bangladesh designed with Eike Roswag, she injects a touch of contemporaneity and strengthens cultures and identities by enhancing local building skills. Her work, recognised with prestigious awards, is extremely valuable because it restores the confidence of craftsmen and encourages them to invest fully in the search for the best possible architectural solutions. This attitude has earned her the admiration of new generations of architects throughout the world.

Chinese architects Wan Shu and Liu Wenyu value the attributes of Chinese cultures, boldly revisiting and including them in their work. The architects encourage the reuse and reappropriation of key elements of vernacular architecture, characterising their work as a dialogue between past and present. As part of this revival of vernacular architecture, they created the Amateur Architecture Studio, a unique space for creation and emancipation. In addition, their architecture is resolutely ecological. They use a wide range of materials, particularly incorporating recycling and traditional techniques, and earth is one of their favourite materials. They appreciate the fact that earth can reduce the ecological footprint of buildings thanks to its hygrothermal qualities and its good carbon balance. Among their most famous earthen projects is the Wa Shan Guesthouse at Xiangshan University and the regeneration of Wencun Village. Wan Shu and Liu Wenyu are creative and disruptive architects who are raising the awareness of the professional community about the value of vernacular architecture (Guillaud 2019).

As we can see, solutions are emerging under the impulse of designers who are seeking to define new codes for contemporary architectural production. These visionary architects draw their inspiration directly from the territories on which they build. They manage to break away from traditional architectural canons to propose new architectures that are full of ecological good sense.

### Preserving and rehabilitating earthen heritage to inspire younger generations

**Fig. 4:**
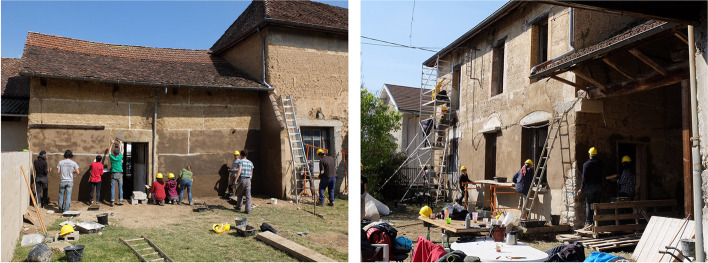
Postgraduate conservation training for young professionals from 15 countries who came to Grenoble in 2021 to specialise in earthen architecture conservation (Source: the authors/CRAterre)

As we have seen, prominent contemporary architects in the revival of earthen architecture have drawn inspiration from vernacular architecture. This heritage is the root of their creativity and the foundation of their projects (De Varine 2005). Some of them grew up in rural areas and saw earthen walls being built from a very young age, while others became interested in traditional earthen buildings out of professional curiosity. Vernacular heritage rarely leaves one indifferent. It is an inescapable source of inspiration that we must preserve at all costs, in both its tangible and intangible dimensions. Indeed, it is not only the beautiful facades, the surprising details and the colourful decorations that must be preserved but also the thoughts and gestures that gave them shape. Earthen architecture is largely understood only on the building site, with a handful of mud - a very changeable material that imposes its own rules. Preserving this earthen heritage that surrounds us is a necessity for reactivating know-how and trying to adapt it to contemporary realities. An architect who has had the chance to build earthen walls in his or her training will be more capable of designing quality earthen buildings that are adapted to the building techniques and reactions of the earth, such as its shrinkage during drying. If he or she is also lucky enough to have worked on the conservation of old earthen structures (Fig. 4), he or she will then be able to avoid the pitfalls of fragile details that may present risks later on. This regular back and forth between new construction and the conservation of old structures is the best school for learning earthen construction techniques. It allows the creation of currently lacking bridges between the vernacular and the contemporary.

### Reviewing the life cycle of buildings

**Fig. 5:**
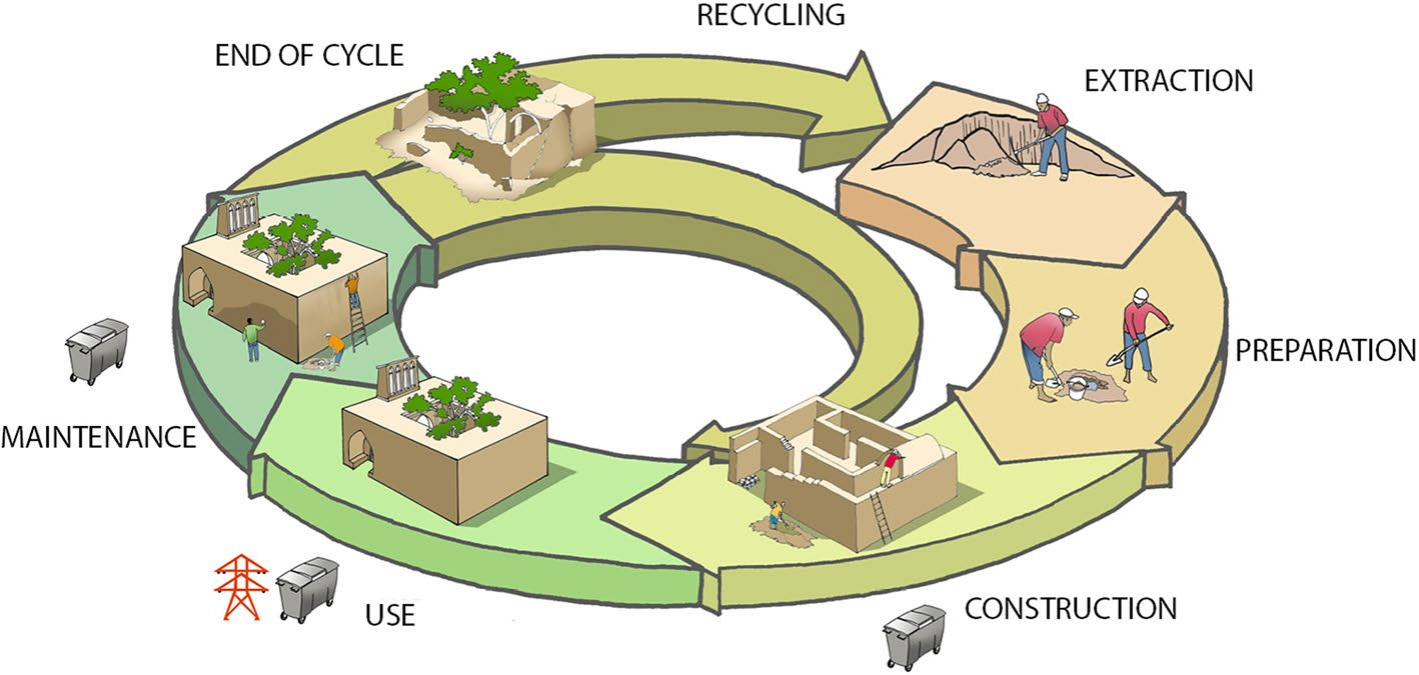
Life cycle of earthen buildings, from earth to earth, with limited energy waste and limited waste production (Source: the authors/CRAterre)

Earthen material is also known to return architecture to less energy-intensive and less environmentally impacting life cycles (Fig. 5). Using raw earth rather than burnt clay bricks already represents a form of energy savings. By paying attention to the many parameters influencing a building’s life cycle, such as the origin of the earth, the way it is prepared and used, and the design of the buildings, the amount of energy required to build can be greatly reduced. However, it is important to be aware that building with earth is not enough to make a project energy efficient. Soil transported over long distances or reworked with sand or other aggregates brought in from afar or an architectural design that is not adapted to the climate or that requires heavy tools for its implementation can quickly weigh down the energy balance of an earthen building. This is why it is necessary to take inspiration from vernacular constructions that represent techniques and architectural forms that are easy to implement with little energy, apart from human energy, which is greatly enhanced.

While in use, an earthen building requires little energy if it is well designed. In the dry, desert areas of Iran, for example, traditional earthen architecture provides thermally comfortable living spaces in all seasons, despite the harsh climate with its extreme temperature ranges. Heating by the sun in winter and cooling by wind towers and qanats in summer provides thermal comfort without any energy consumption. Earth has the well-known property of regulating humidity and thus the comfort of interior spaces (Moriset et al. 2018). Electric heaters and air conditioners are now used to achieve the same result but come with high costs and environmental pollution.

At the end of an earthen building’s life cycle, the soil can be reused or left on the ground without the risk of pollution. Our^2^ extensive conservation activities at archaeological sites have shown that soil from walls that collapsed thousands of years ago is still suitable for construction. It is possible to moisten it and remould bricks that have the same mechanical properties and colour as those moulded thousands of years earlier.

By aiming for the most efficient life cycle possible in terms of energy savings and reduced pollution, we return to using unprocessed local resources, mastered in the construction and maintenance phases by the local populations, to build architectures offering real climatic comfort. Vernacular architecture offers rich lessons in this respect.

### Rewarding efforts

Earthen architecture also owes its renaissance to internationally recognised architects who have highlighted its ecological and aesthetic qualities. When winners of major international architectural prizes such as Renzo Piano, Wang Shu, Herzog & de Meuron, Anna Heringer, Patrice Doat, and Francis Kéré, bring this material to the highest level, the whole profession is challenged and looks at this “material for the poor” (Fathy 1973) differently.

Specialised awards have also played an important role in the development of earthen architecture. The first national award for earthen architecture was launched in 2013 in France. It was transformed into an international award on earthen architecture in 2016 (Terra Award), under the aegis of CRAterre/ENSAG alongside various partners. The Terra Award has led to the international recognition of the qualities of earthen architectures through a travelling exhibition. These awards have helped identify many committed architects and builders on all continents, with 300 applications received for the first edition. The awards have also enhanced the prestige of the owners and builders who have often had to fight to build with earth. Ultimately, these specialised awards have a real impact and, according to many earthen construction specialists, coincide with an increase in demand for technical advice on the part of builders and contractors. These dynamics continue with the Terra Award Sahel+ (2019) and the launch of the second Terra Award (2021).

Specialised professionals are multiplying in number, and the desire for eco-responsible architecture among various actors is taking shape. However, we must remain vigilant to ensure that fashions and trends do not undermine the quality of construction. Recently, there has been a craze for the rammed earth technique due to its aesthetic qualities, which stem from its implementation in successive layers that are immediately recognisable. Unfortunately, this technique is sometimes parachuted in for free use in unsuitable contexts or construction projects. Training efforts must be intensified to ensure the quality of architectural production and enhance the diversity of construction techniques and typologies offered by raw earth.

## Encouraging research

In the field of earthen architecture, researchers have shown an initial interest in heritage. As conservation projects of earthen sites, especially archaeological ones, multiplied across the world (Gandreau 2017), the first international conferences of experts were organised to allow an exchange of experiences and to consider the subject from a theoretical point of view. The first conference took place in Yazd, Iran in 1972 and was organised by ICOMOS-Iran. They were called TERRA conferences, then TERRA congresses, and are still held once every 4 years. The 1973 oil crisis also gave rise to research movements pushing for alternatives in architecture, particularly in building materials.

In 1983, the fourth international TERRA meeting in Lima (Peru) was supported not only by ICOMOS but also by UNESCO, ICCROM and CRAterre-EAG. This marked the beginning of institutional cooperation to create a long-term programme combining research, education, planning, application and dissemination. This led to the launch in 1989 of the international cooperation project GAIA, which brought together ICCROM and CRAterre-EAG. It was followed in 1998 by the TERRA project (1998–2005), which the Getty Conservation Institute participated in.^3^ These international collaborations were a coherent effort to “promote a consistent scientific approach” (Alva et al. 1990) for conservation, research and cooperation in the area of earthen architecture conservation and promotion.

In 2007, international cooperation took a further step forward when, in its 31st session (New Zealand, 2007), the World Heritage Committee approved the launch of an integrated World Heritage Programme for Earthen Architecture (2007–2017) as “an integrated approach based on different lessons learnt” (Monteil 2014).

Additionally, at the beginning of the 21st century, the tendency was to affirm that “earthen architecture has become a discipline on its own”^4^ (Houben 2011). Nevertheless, much remains to be done if this discipline is to be recognised in the same way as other long-established disciplines (Correia et al. 2016).

## Consolidate and disseminate knowledge

### Train at all levels

Efforts to disseminate earthen architecture techniques are largely fuelled by scientific research. Thus, the work carried out by CRAterre on world architectures led to the publication in 1989 of the Earth Construction comprehensive guide available in 5 languages (French, English, Spanish, Russian and Arabic). Since then, several scientific research programmes dedicated to earth have been carried out in architecture and engineering schools around the world. They provide knowledge on earthen architecture throughout the life cycle of buildings. Specific programmes on vernacular architecture, such as the VERSUS^5^ programme, have capitalised on the wealth of useful knowledge and know-how for the benefit of training institutions.

### Development of degree courses

The first post-master’s course in earthen architecture at the Grenoble School of Architecture (ENSAG), France, was organised in 1984. Today, there are nearly 350 professionals from over 50 countries who have graduated from the programme. Many of them have become university educators in their home countries. Together, they constitute a large part of the active membership of the UNITWIN network of the UNESCO Chair on Earthen Architecture, which includes 44 member institutions spread over 4 continents.^6^

### Tools for vocational training

To sustain this dynamic, several multi-annual projects have created educational material for academic and professional dissemination. One example is the three-year European “earth today, earthen plasters” project (2003–2006) implemented under the Leonardo da Vinci Programme by 14 partners from six European countries (Germany, Bulgaria, France, Greece, Poland, and the United Kingdom). As part of the programme, universities, research centres, associations and companies cooperated to create a vocational training unit with the objective of developing basic knowledge on earthen materials.

More recently, the European project “Pirate” aimed to provide instruction and resources to train and evaluate capacities in earthen construction. This three-year project (2012–2015) dedicated to professional training brought together 18 partners from 8 European countries and produced training standards recognised at the European level.

The Ibero-American network of earthen architecture professionals (RedPROTERRA) is also very active in producing educational materials, now available for free download. These include publications on earth analysis, quality control and construction techniques.

We should also note the growing interest of Middle East countries, the cradle of earthen architecture on the planet, in establishing university courses to prepare young professionals for conserving earthen heritage and designing contemporary earthen architecture. This interest is part of these countries’ post-oil vision to which they must adapt.

University and professional training courses are therefore multiplying worldwide to meet the pressing demand for eco-responsible architecture. The pedagogical tools needed to set up such programmes are increasingly easy to find.

### Innovative training models

**Fig. 6:**
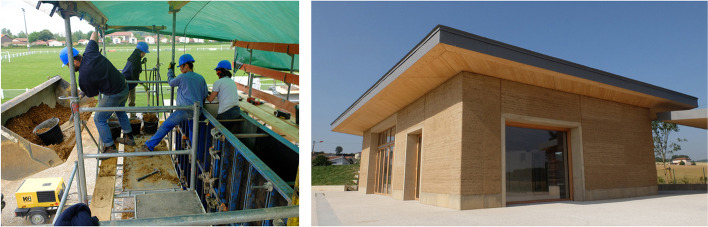
“House for all” in the commune of Four, France, built in 2018 by students from the Grenoble School of Architecture as part of the design/buildLAB supervised by Keith and Marie Zawistowski (Source: the authors/CRAterre)

Learning outside the classroom is an educational option that has been recognised and acclaimed since 1993 by the Rural Studio^7^ programme of Auburn University in Alabama, USA. The programme developed a model of hands-on training while providing services to the community. Keith and Marie Zawistowski in turn exported this teaching model to the French countryside. Together, they supervise the design/buildLAB,^8^ which has become a teaching, research and community service initiative of the Grenoble School of Architecture’s LabEx^9^ AE&CC^10^ laboratory. Keith and Marie Zawistowski’s work is rooted in the territory and revitalises ancestral techniques while experimenting with large-scale technical innovations (Fig. 6). In this context offered to master’s students in architecture, earth reveals its intrinsic capacity to be educational because it does not lie about its technical capacities and is appropriable by construction novices. Constructions are carried out at the request of local authorities in collaboration with local companies, which also learn to build differently through these methods.

### Networking and public investment in earth construction

The rebirth of earthen architecture requires joint efforts between public institutions and private operators, which are beginning to be structured not only in Europe but also on other continents. In China, for example, architect Mu Jun, concerned with improving housing in rural and economically deprived areas of China, has embarked on an innovative path. Starting with a research project launched in 2011 in collaboration with CRAterre-ENSAG in Macha, Gansu Province, he has led a renewal of rammed earth building using locally available resources. The results of his research convinced the Ministry of Housing and Urban and Rural Development in 2018 to support the construction of 190 houses and public institutions in 11 regions. These projects served as training platforms for young professionals. They enabled 400 architecture students to participate in numerous technical training courses in situ within the framework of the programme. Through these pragmatic and useful experiences, students are able to reappropriate ancestral and rural Chinese cultures.

This enthusiasm for earth is leading to the creation of a network of project leaders. On an international scale, there is the UNESCO Chair in Earthen Architecture held by CRAterre/AE&CC/ENSAG,^11^ which supports the development of research and education on a global scale; ICOMOS-ISCEAH,^12^ which aims to conserve earthen heritage; Proterra,^13^ a network of professionals from Latin America and the Iberian Peninsula; FACT Sahel,^14^ a network in Sahelian Africa; and Mediterra in the Mediterranean.

Professional networks also operate at the national level in Germany (Dachverband Lehm^15^), the United Kingdom and Ireland (Ebuki^16^), Portugal (Centro da Terra^17^), France (Asterre^18^) and Australia (Earth Building Association of Australia^19^).

An increasing number of local authorities are also following this approach. As early as 2001, the network of earthen cities in Italy (Citta della Terra Cruda^20^) was created to promote vernacular heritage. Since 2002, the Rhône-Alpes region in France has also supported the development of raw earth architecture in its various sustainable development programmes. As a result, some municipalities have been mobilised to carry out contemporary architecture projects using raw earth, with the support of social housing owners committed to eco-construction. The revision of regulations on heritage is also part of these programmes and aims to curb bad practices in the conservation of earthen buildings.

Local authorities are therefore the driving force behind these efforts and are in need of guidance. Indeed, they are often confronted with the mandate to protect earthen heritage but are ill-equipped to manage it. Several local departments are concerned: heritage, urban planning, housing, environment, tourism, and technical services. To meet this demand, an orientation guide for the management of rammed earth in the Rhône-Alpes-Auvergne region, where nearly 22% of municipalities are engaged in with rammed earth projects, was published (Gandreau et al. 2018), and training programmes, which were launched in 2018, are continuing with national support.

### Supporting the industrial production of earthen building materials

For several decades, European companies have been committed to facilitating earth construction by selling earth-based materials. As early as 1984, the German company Claytec,^21^ which is still operational today, sold unfired earth bricks and clay panels cast on a woven straw support, as well as bagged earth. This practice is expanding in Europe with the appearance of numerous companies supplying ready-to-use earth in response to the demand for healthy construction materials. These ready-made products encourage the appropriation of earth by nonexperts, which is good, but they also allow earth to be trucked thousands of kilometres away, which is counterproductive. Training craftspeople to work with local soil or avoiding the geographical dispersion of these soil-based products remains the best option.

By proposing technical advances, several companies are also involved in the dissemination of earthen architecture. As early as 1996, Martin Rauch supported the prefabrication of rammed earth elements to speed up building projects and reduce drudgery. In 2010, the French company Caracol, supported by various players in the French sector, explored poured earth based on a formulation close to rammed earth. While technical and technological advances correspond to performance needs, ecological priorities are becoming increasingly challenging for companies.

The climate emergency requires new ways of working that involve greater numbers of people than ever before. Recently, the question of the large-scale production of eco-responsible architecture has been considered in relation to the problem of managing tens of millions of tons of earthen debris that are excavated from urban subsoils every day and stored at great expense outside of cities. As part of Cycleterre,^22^ an innovative project supported by the European Union, companies, project owners and project managers are experimenting with, standardising, and organising production lines and training to transform the “waste” subsoil of Paris into building elements such as panels, bricks and earth plasters. This urban production of local building materials has the advantage of providing employment and minimising pollution by limiting the round trips of trucks taking soil out of the city and then importing industrial materials produced far from urban areas.

The economic issues related to earthen architecture are still in full development mode. Earthen construction outside the self-build context is rarely competitive if one compares only the costs of wall construction per m^2^. The high labour required to erect earthen buildings causes significant additional costs in most economic conditions, but these extra costs feed and strengthen the local economy by providing job opportunities. For earth to be a competitive material, the cost of buildings must be calculated on a whole-life cycle basis because once in use, these structures help cut energy costs for comfort, and their maintenance is often lower than that of surfaces that require cyclical cleaning or repainting. Interior earthen plasters do not flake, do not discolour and are very easy to repair with a sponge and water. It is on the basis of these considerations that earthen construction is now being revived. Clients are willing to pay more for a building that will cost them much less in the long run. The health dimension is also taken very seriously in economic terms, as unhealthy materials are very expensive for national healthcare systems. Most industrial materials available on the market today generate greenhouse gases from extraction to construction and then emit volatile organic compounds that are harmful to residents. This is an additional incentive to return not only to earth but also to all organic and geological resources to preserve the economic health of all regions and the physical health of their citizens.

## Conclusion

The gradual industrialisation of building material production is irreversible, but the return to endogenous knowledge is critical. The disdain for earthen architecture is becoming less pronounced. Vernacular architecture, and earthen architecture in particular, seems to be a voice of wisdom and source of knowledge that we have been missing for the last 50 years. The old earthen buildings of our planet are an excellent research path for helping us change course and rethink our relationship to building as well as our relationship to the different forms of life on earth. Analysing heritage means listening to what our ancestors have to say and forgetting the clean slate approach. Even if our mindset has evolved, the will of our predecessors to make the most of the available resources is again relevant, and their achievements have revealed the way forward to many upcoming builders, architects and government authorities. Although earthen materials still face restrictive legislation and standards that do not allow their use in many countries, they are gradually being rehabilitated. Several states on different continents have adopted regulatory measures to accompany the renewal of earthen architecture.

Therefore, we must ask: can earthen architectural heritage save human beings? We believe so—earth has demonstrated its effectiveness in many parts of the world. The pioneers of the earthen architecture revival rediscovered this amazing material after the first oil crises of the 1970s. Alternative solutions often emerge in times of emergency, and the multiple crises we face today, particularly regarding the climate, require changes in the way we build. Transport restrictions due to the COVID-19 pandemic are already causing shortages and, consequently, price escalations for many industrial building materials. Earth is not affected by these fluctuations. Future building regulations could make it mandatory for actors in the building sector to use local materials, particularly in the European Union and China. It is therefore time to start: earth is an amazing material that will help us find ways to initiate a real change in our relationship with architecture.

## Data Availability

Not applicable.

## Footnotes

1 These architectures and the way these populations manage the territory that hosts them are particularly outstanding with regard to the United Nations 17 Sustainable Development Goals (SDG): They help avoid hunger (SDG2), provide healthy food/building products that are responsibly produced in a healthy environment (SDG3 and 12), ensure a fair and equal distribution of resources (SDG10), do not encourage climate disruption (SDG13), respect life on land (SDG15), and contribute to peaceful relationships between humans (SDG16).

2 The authors have been working for over 20 years on the conservation of prestigious earthen archaeological sites, particularly in the Arabian Peninsula, Iran and Central Asia.

3 https://www.getty.edu/conservation/our_projects/field_projects/terra/

4 Title of an article by Hugo Houben, one of the founders of CRAterre, who unfortunately passed away in January 2021.

5 www.esg.pt/versus

6 CRAterre: UNESCO Chair on earthen architecture and sustainable development; see http://www.craterre.org/enseignement:chaire-unesco/?new_lang=en_GB

7 http://ruralstudio.org/about

8 http://www.designbuildlab.org

9 LaBex stands for “Laboratoires d’Excellence”, that is to say, a group of excellent research laboratories.

10 AE&CC: Architecture, Environment & Constructive Cultures research Unit

11 http://www.craterre.org/enseignement:chaire-unesco/?new_lang=en_GB

12 ICOMOS ISCEAH – International Scientific Committee on the Conservation of Earthen Architectural Heritage, see http://isceah.icomos.org

13 https://redproterra.org/pt

14 https://www.factsahelplus.com

15 https://www.dachverband-lehm.de

16 See the Facebook account of Earth Building UK and Ireland (https://www.facebook.com/earthbuildinguk).

17 https://centrodaterra.org

18 https://www.asterre.org

19 See the official website of the Earth Building Association of Australia (https://www.ebaa.asn.au).

20 https://www.terracruda.org

21 https://www.claytec.de/en

22 https://www.cycle-terre.eu
